# A survey of methods and tools to detect recent and strong positive selection

**DOI:** 10.1186/s40709-017-0064-0

**Published:** 2017-04-08

**Authors:** Pavlos Pavlidis, Nikolaos Alachiotis

**Affiliations:** Institute of Computer Science, Foundation for Research and Technology-Hellas, 70013 Crete, Greece

**Keywords:** Positive selection, Selective sweep

## Abstract

Positive selection occurs when an allele is favored by natural selection. The frequency of the favored allele increases in the population and due to genetic hitchhiking the neighboring linked variation diminishes, creating so-called selective sweeps. Detecting traces of positive selection in genomes is achieved by searching for signatures introduced by selective sweeps, such as regions of reduced variation, a specific shift of the site frequency spectrum, and particular LD patterns in the region. A variety of methods and tools can be used for detecting sweeps, ranging from simple implementations that compute summary statistics such as Tajima’s D, to more advanced statistical approaches that use combinations of statistics, maximum likelihood, machine learning etc. In this survey, we present and discuss summary statistics and software tools, and classify them based on the selective sweep signature they detect, i.e., SFS-based vs. LD-based, as well as their capacity to analyze whole genomes or just subgenomic regions. Additionally, we summarize the results of comparisons among four open-source software releases (SweeD, SweepFinder, SweepFinder2, and OmegaPlus) regarding sensitivity, specificity, and execution times. In equilibrium neutral models or mild bottlenecks, both SFS- and LD-based methods are able to detect selective sweeps accurately. Methods and tools that rely on LD exhibit higher true positive rates than SFS-based ones under the model of a single sweep or recurrent hitchhiking. However, their false positive rate is elevated when a misspecified demographic model is used to represent the null hypothesis. When the correct (or similar to the correct) demographic model is used instead, the false positive rates are considerably reduced. The accuracy of detecting the true target of selection is decreased in bottleneck scenarios. In terms of execution time, LD-based methods are typically faster than SFS-based methods, due to the nature of required arithmetic.

## Background

Evolution by natural selection is based on a simple principle: traits that increase the chance of survival and reproduction have a higher tendency to be transmitted to the next generation. The beauty of evolution by natural selection is in the simplicity with which adaptation is achieved over time. The definition is universal since it does not distinguish between the various forms of natural selection, such as positive selection, negative selection, balancing selection, and frequency-dependent selection, neither does it depend on the fitness landscape nor on the way that a population explores it. In addition, it does not differentiate between single-locus and multi-loci traits, and it does not assume any independence between loci or any form of epistasis. The generality of the natural selection concept, however, yields the detection of traits that have contributed to the adaptation of organisms a rather challenging task. The definition itself is intuitive, clear, and well-understood. Yet, it does not provide any means on how to detect adaptive traits. Therefore, research has predominantly focused on the various forms of natural selection (e.g., positive, negative, balancing etc.) in order to understand and describe them, as well as to provide the means and tools to detect them.

Positive (or directional) selection is among the most extensively studied forms of selection, occurring when an allele is favored by natural selection. In that case, the frequency of the beneficial/favored allele increases over time, potentially becoming fixed in the population (substituting the non-beneficial one) when the effective population size ($N_e$) is large and back mutations occur infrequently. In a seminal study, Maynard Smith and Haigh [1] showed that when a beneficial allele substitutes a neutral allele, the frequencies of closely linked *neutral* alleles change as well. Those alleles that were originally linked to the benefical allele increase in frequency, whereas the remaining—non-linked—ones decrease in frequency. Maynard Smith and Haigh [1] coined the term ‘hitchhiking’ to describe this effect, because a neutral allele can get a lift by a closely linked beneficial allele. They also showed that heterozygosity at a linked locus is proportional to *c*/*s*, where *c* is the fraction of recombination rates between the neutral and the beneficial loci, while *s* is the selection coefficient of the beneficial allele. The fraction of recombination rate *c* delimits the effect of hitchhiking locally in the genome. At distant locations, recombination breaks the physical linkage to the beneficial allele and therefore distant regions evolve independently of the selective sweep. Interestingly, the motivation of Maynard Smith and Haigh to study the hitchhiking effect came from an observation by Lewontin [2], that the extent of enzyme polymorphisms is surprisingly constant between species of very different effective population sizes (see Box).

**Table:** Effective population size

The concept of the Effective Population Size was firstly introduced by Sewall Wright in 1931 [3]. Wright introduced *N* (the symbol $N_e$ is mostly employed today instead) to describe the size of a diploid breeding population, which is smaller than the total number of individuals of all ages. He shows that population size fluctuations brings the effective *N* closer to the smaller actual population size. Also, the unequal numbers between males and females reduce the effective *N*. Finally, variations on the offspring numbers also reduce the effective population size. The effective population size is almost always smaller than the actual population size. A notable exception is the case of seedbanks, where the effective population size (hidden in forms of seeds) may be orders of magnitudes greater than the actual number of developed organisms [4, 5].	

Assuming that the $N_e$ is sufficiently large, Maynard Smith and Haigh [1] showed that the hitchhiking effect can have a considerable aggregate effect on the reduction of the polymorphism levels within populations. This result is roughly correct for finite population sizes as well [6, 7]. Therefore, the effect of $N_e$ on the polymorphism level would be buffered by the hitchhiking effect, and differences on the heterozygosity between populations of very different effective population sizes will not be as significant as predicted by neutrality:

1 $$\begin{aligned} H = 4 N_e u / ( 1 + 4 N_e u), \end{aligned}$$

where *u* is the mutation rate, and *H* is the amount of heterozygosity. Using the wording from Maynard Smith and Haigh: “If *H* lies between 0.1 and 0.5, then $N_e$ lies between 0.028 and 0.25 u^−1^, and it is not plausible that the effective population sizes of all species lie within such narrow limits”.

Due to its simplicity, as well as the potential to generate testable hypotheses, the hitchhiking effect motivated the study of the various signatures that a beneficial allele leaves locally on the genome upon fixation. A first prediction is the reduction of the polymorphism level locally on the genome. Because of this property of the hitchhiking effect to sweep the neutral polymorphisms in the neighborhood of a beneficial mutation, the term ‘selective sweep’ has been coined. In fact, according to the hitchhiking model, genomic regions with low recombination rates (per base pair and per individual) exhibit less diversity. In *Drosophila*, studies have confirmed this prediction in regions of reduced recombination. In *D. melanogaster*, Aguade et al. [8] studied the *yellow-achaete-scute* complex located in a region of reduced crossing over, close to the telomere, and observed that the level of diversity is reduced in relation to regions of normal crossing over, consistently with the hitchhiking effect hypothesis. In *D. ananassae*, Stephan and Langley [9] also reported reduced genetic variability in a region of reduced recombination rate. They studied the *vermilion* locus in the centromeric region, concluding that their results are consistent with the hitchhiking model. A second signature that hitchhiking leaves on the genome is a particular shift of the Site Frequency Spectrum (SFS) [10, 11]. Specifically, an increase of high- and low-frequency derived variants is expected in the proximity of the beneficial mutation. A third signature is associated with the level of Linkage Disequilibrium (LD). As shown by [12, 13], the LD levels remain high at each side of the beneficial mutation, and drop dramatically for loci across the beneficial mutation. These three signatures motivated the design of several tests to detect genomic regions subject to genetic hitchhiking.

Testing for the effect of genetic hitchhiking, typically referred to as selective sweep detection, is achieved by a variety of means, ranging from simple summary statistics to standalone software tools. These tests vary on the signatures they detect, such as SFS- vs. LD-based methods, and/or on the applicability of the implementations, such as genome-wide vs. subgenomic regions.

Recently, several excellent surveys on detecting selective sweeps have been published. Malaspinas [14] focused on methods that detect selective sweeps in ancient DNA (aDNA) samples and time series data. The author presents an extensive table of methods, providing brief guidelines about when to use each approach, the inference each method is able to perform, their assumptions, as well as studies and organisms they have been applied on.

Crisci et al. [15] reviewed several widely-used approaches to detect recent and strong positive selection, such as SweepFinder [16], SweeD [17], OmegaPlus [18], and iHS [19]. The study mostly focuses on the type I and II error of the methods, the effect of population parameters, such as population substructure and/or population size, and the length of the sequenced region. The authors performed simulations to demonstrate the efficiency of the different methods, finding that LD-based methods outperform other methods in both equilibrium and non-equilibrium evolutionary scenarios.

Vitti et al. [20], in an extended review, reported ideas and concepts that have been used to detect selection on a macroevolutionary or microevolutionary scale. They go beyond the classical model of selection (complete or ongoing selective sweeps) and discuss more complex models of natural selection, i.e., soft selective sweeps or selection on polygenic traits. Finally, they report a list of the most important genes found to be evolved under selection.

Pool et al. [21] review the challenges posed by new generation sequencing data, particularly with respect to data quality and missing values. They assess the challenges of analyzing polymorphisms on the whole-genome scale, and the potential analyses that can provide insights into the inference of population genetics parameters using whole-genome data.

In this review, we survey methods and tools that can be used to detect recent and strong positive selection, or equivalently, so-called ‘hard’ selective sweeps. We provide insights into performance issues of the methods, as well as their accuracy to detect the target of selection in natural populations. The remaining of this survey is organized as follows: in section "Sweep footprints and problems caused by demography", we describe the three different signatures of a selective sweep, and discuss the problems introduced in the detection process by neutral demographic events. In "Methods and tools" we present summary statistics and stand-alone software tools. We classify them based on the signature they detect and the applicability on whole genomes or subgenomic regions. Evaluation results regarding sensitivity, specificity, and execution times are presented in section "Evaluation". The subsequent section "Detection of soft sweeps" presents methods for detecting soft selective sweeps, while the "Discussion" section focuses on interpretation, performance, and efficiency issues.

## Sweep footprints and problems caused by demography

### Detecting sweeps based on diversity reduction

The most striking effect of genetic hitchhiking is the reduction of the polymorphism (diversity) level. Maynard Smith and Haigh [1] predicted the reduction of heterozygosity as a consequence of the hitchhiking effect in large (infinite) populations, immediately after the fixation of the beneficial mutation. After the completion of the hitchhiking effect, when the beneficial mutation has been fixed, neutral variation will start to accumulate again on the genomic region and heterozygosity will increase. A prediction of the hitchhiking effect is that in genomic regions with reduced recombination rate per physical distance, the amount of diversity decreases if the hitchhiking effect is recent. Subsequent studies [7–9, 22–25] confirmed this prediction for *D. melanogaster*, *D. simulans*, and *D. ananassae* species. A similar prediction, however, holds for background selection [26] as well. More specifically, if neutral variants are linked to a strongly deleterious mutation, the level of polymorphism also deteriorates, since the deleterious mutation is gradually removed from the population. The amount of polymorphism reduction depends on the selection coefficient of the deleterious mutation [27]. For example, there is no effect when the linked deleterious mutation is lethal, since it is being directly removed from the population. Even though both evolutionary forces predict the reduction of the diversity level, it has been demonstrated [28] that, in a hitchhiking model, the estimated level of diversity, $\hat{\theta }$, is negatively correlated with $\hat{\theta }/\rho$, where $\rho$ is the recombination rate, whereas in a background selection model, the estimated level of diversity is positively correlated with the same quantity (see also [29] for a review).

### Detecting sweeps based on the SFS

**Fig. 1:**
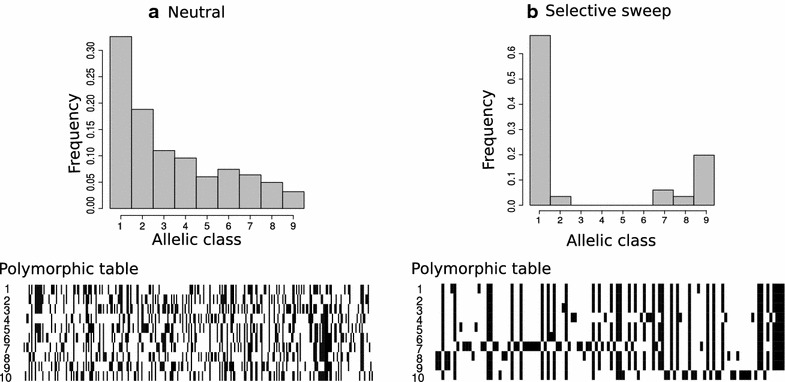
The SFS signature of a selective sweep compared to the neutral SFS. In the polymorphic table,* black cells* denote derived alleles, whereas the white cells denote ancestral alleles. Each* column* in the polymorphic table represents a SNP. Monomorphic sites have been excluded.** a** Neutral SFS and its respective polymorphic table.** b** SFS after a selective sweep and its respective polymorphic table

The studies by [10, 11] showed that a selective sweep triggers a shift of the SFS toward high- and low-frequency derived variants. This is attributed to the fact that neutral variants that are initially linked to the beneficial variant, increase in frequency, whereas those ones that are initially not linked to the beneficial variant decrease in frequency during the fixation of the beneficial mutation. Figure 1 illustrates the shift of the SFS after a selective sweep and the corresponding polymorphic table.

A breakthrough on detecting selective sweep approaches was the test proposed by [30], known as the Kim and Stephan test for selective sweeps. They developed a composite-likelihood-ratio (CLR) test to compare the probability of the observed polymorphism data under the standard neutral model with the probability of observing the data under a model of selective sweep. For the selective sweep model, and for each value of the selection intensity ($a = 4 N_e s$), where *s* is the selection coefficient, the test calculates the probability to observe the data and reports the value of *a* that maximizes the CLR. Thus, besides the detection of the location of the selective sweep, the Kim and Stephan test is able to estimate the strength of selection as well. The Kim and Stephan test was the first to implement a CLR test on sweep detection, and it has been used to detect selection on candidate loci [31, 32]. It adopts, however, several oversimplified assumptions. First, the neutral model was derived by an equilibrium neutral population, i.e., a population with constant population size. Second, the selection model was derived by Fay and Wu’s model [11], where only the low- and the high-frequency derived classes are assumed. Concerning the execution of the Kim and Stephan test, run time and memory requirements are extensively large, yielding the approach not suitable for genome-scale detection of selective sweeps.

### Detecting sweeps based on LD

**Fig. 2:**
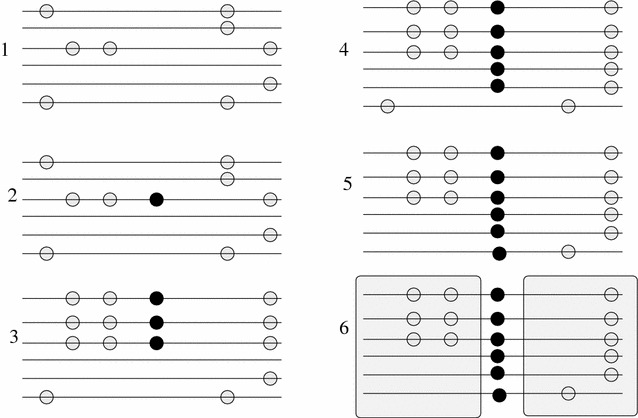
The LD signature around a selective sweep. Assume a population with neutral segregating variation (*1*). A beneficial mutation occurs (shown as a* black* allele) in subfigure (*2*). Since the mutation is beneficial, its frequency will increase in the population. Neutral variants that are linked to the beneficial mutation will hitchhike with it (*3*). Due to recombination, mutations from a neutral background will get linked with the beneficial mutation (*4*,* 5*). Finally, the selective sweep completes (*6*). The LD pattern that emerges from such a process is the elevated LD on each side of the beneficial mutation and the decreased LD for SNPs that are on different sides of the beneficial mutation

The third signature of a selective sweep consists of a specific pattern of LD that emerges between SNPs in the neighborhood of the target site for positive selection. Upon fixation of the beneficial mutation, elevated levels of LD emerge on each side of the selected site, whereas a decreased LD level is observed between sites found on different sides of the selected site. The high LD levels on the different sides of the selected locus are due to the fact that *a single* recombination event allows existing polymorphisms *on the same side of the sweep* to escape the sweep. On the other hand, polymorphisms that reside on different sides of the selected locus need a minimum of two recombination events in order to escape the sweep. Given that recombination events are independent, the level of LD between SNPs that are located on different sides of the positively selected mutation decreases. Figure 2 shows an example of the LD patterns emerging after a sweep.

The LD-based signature of a selective sweep was thoroughly investigated by Kim and Nielsen [12]. In this study, they introduced a simple statistic, named $\omega$-*statistic*, that facilitates the detection of the specific LD patterns that emerge after a sweep. For a window of *W* SNPs that is split into two non-overlapping subregions *L* and *R*, with *l* and $W-l$ SNPs, respectively, the $\omega$-*statistic* is computed as follows:

2 $$\begin{aligned} \omega = \frac{\left({l \atopwithdelims ()2} + {W-l \atopwithdelims ()2}\right)^{-1}\left(\sum _{i,j\in L}r_{ij}^2 + \sum _{i,j\in R}r_{ij}^2\right)}{\left(l(W-l)\right)^{-1}\sum _{i\in L, j\in R}r_{ij}^2}. \end{aligned}$$

Jensen et al. [33] evaluated the performance of the $\omega{\text{-}}statistic$ in terms of the capacity to separate between neutral demographic models and selective sweeps, and showed that the $\omega{\text{-}}statistic$ accurately detects the targets of positive selection for demographic parameters relevant to natural non-equilibrium populations, such as the cosmopolitan population of *D. melanogaster*.

### The role of demography in selective sweep detection

**Fig. 3:**
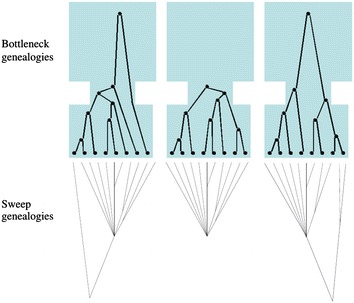
Bottleneck demographic scenarios (*top panel*) may result in similar genealogies to a selective sweep (*bottom panel*). Both models may produce very short coalescent trees. As we move from the selection site, selective sweeps produce genealogies with long internal branches. Similarly, bottlenecks may produce genealogies with very long internal branches if the ancestral population size is large

Demography introduces severe challenges on the detection process for positive selection due to its confounding nature regarding the signatures of genetic hitchhiking. Selective sweep detection becomes feasible mainly due to two factors: (a) the fixation of the beneficial mutation, and b) the fact that coalescent events occur at a higher rate in the presence of a sweep than they do in its absence. It is these two factors, along with *recombination events*, that generate the specific signatures of a selective sweep, enabling us to detect traces of positive selection in genomes. However, additional factors can also trigger a high rate of coalescent events, leading to the generation of similar (to a selective sweep) signatures in the genome, and thus misleading current selective sweep detection approaches. For instance, assume a bottleneck event that is characterized by three phases: (a) a recent phase of large effective population size, (b) a second phase, prior to the first one, of small population size, and (c) an ancestral one of large population size. It is due to the decrease of the effective population size in the second phase that a high rate of coalescent events occur, thus raising the possibility of observing a large number of coalescent events in a relatively short period of time. Furthermore, if the second phase is not too severe, lineages can escape the bottleneck, passing to the ancestral phase of large effective population size, and therefore requiring more time to coalesce. In a recombining chromosome, genomic regions that have witnessed a massive amount of coalescent events during the bottleneck phase may alternate with genomic regions with lineages that have escaped the bottleneck phase (Fig. 3). Such alternations can generate SNP patterns that are highly similar to those generated by a selective sweep, yielding the detection process very challenging, if not unfeasible [34].

It is well known that certain demographic scenarios generate spurious SNP patterns that resemble a selective sweep. Yet, it is generally believed that, unlike the localized effect of a selective sweep, neutral demographic changes generate genome-wide patterns. This idea of ‘local sweep effects’ vs. ‘global demographic effects’ has been extensively used to regulate the demography-induced false positive rates [16, 17, 35]. In SFS-based sweep scans, this idea translates to a two-step computational approach that entails the initial estimation of an average, genome-wide SFS (background SFS) followed by a detection step, for those genomic regions that fit the selection model but not the background SFS. An issue with such an approach, however, is that it does not take into account the variation of the SFS in different regions of the genome, and it assumes an approximately uniform behavior of the SFS along a recombining genome. This is not the case for demographic models, such as bottlenecks, which generate great variance along a recombining chromosome [34, 36–38]. Therefore, under certain bottleneck demographic scenarios, there can be neutral-like genomic regions, as well as sweep-resembling ones, regardless of the actual existence of a selective sweep. Since both recombination and the alternation of genealogies along a recombining chromosome are stochastic, it is highly challenging to determine which genealogies are shaped by the neutral demographic process and which genealogies are shaped by the action of positive selection at a certain location in the genome. Current approaches are not able to completely overcome the confounding effect of bottlenecks on positive selection in recombining chromosomes, therefore users should be careful when interpreting results of selective sweep scans. It should be noted however, that, several tools, such as SweepFinder, SweepFinder2, SweeD, and OmegaPlus, and/or the deployment of the demographic model as the null model, contribute to alleviating the problem generated by the confounding effects of demography.

Demography not only affects the False Positive Rate (FPR) of the detection methods, or our ability to distinguish it from selective sweeps, but additionally represents an obstacle in the detection process. This derives from the fact that the SNP patterns which emerge from the combined action of *demography and selection* are unknown. For instance, the SFS-based tools SweepFinder and SweeD (presented in a following section), assume that if a lineage escapes the selective sweep due to a recombination event, then, prior to the sweep, its frequency is given by the neutral (or background) SFS. This is valid if the selective sweep has occurred in a constant-size population. If, however, the population has experienced population size changes (or other demographic events such as migrations), this assumption does not necessarily hold.

Given the difficulties that bottlenecks pose on identifying accurately the footprints of selection, it is unfortunate (even though expected) that most natural populations have experienced bottlenecks during their evolutionary history. For example, the European population of *D. melanogaster* experienced a severe bottleneck about 15,800 years ago, when the European population diverged from the African population. The duration of the bottleneck was about 340 years and the effective population size during the bottleneck was only 2200 individuals [39]. Regarding the demography of human populations, the proposed models suggest several bottleneck (founder) events and interactions (gene flow) between subpopulations [40]. Domesticated animals have also experienced a series of bottleneck events during the domestication process. Using only mtDNA and the Approximate Bayesian Computation methodology, Gerbault et al. [41] report that goats have experienced severe bottleneck events during their domestication. Approximate Bayesian Computation was also used to provide insights into the demographic history of silkworm [42]. Using 17 loci in the domesticated silkworm, they reported that the most plausible scenario explaining the demographic history of silkworm comprises both bottleneck and gene flow events [42].

## Methods and tools

### Summary statistics

Summary statistics are inexpensive calculations on the data, typically implemented following a sliding window approach where the window slides along the genome with a fixed step. Simpler statistics such as Tajima’s D or the SNP count do not require sequencing, but only SNP calling, whereas LD-based ones, like counting the number of haplotypes or measuring haplotypic heterozygosity require sequencing prior to scanning the genomes. Several summary statistics serve as neutrality tests due to the fact that their distributions differ distinctively between neutrality and the presence of strong positive selection.

Relying on Tajima’s D, Braveman et al. [10] were able to detect genomic regions affected by recent and strong positive selection in simulated datasets, as well as to demonstrate that regions of low genetic diversity and low recombination rate (e.g., around centromeres or at telomeres) are not compatible with a simple hitchhiking model. Since then, Tajima’s D has been deployed in numerous studies as a neutrality test to detect selection [43–49]. This summary statistic captures the difference between two estimates of the diversity level $\theta = 4 N_e \mu$, where $\mu$ is the mutation rate. The first estimate, $\pi$, is based on the number of pairwise differences between sequences, while the second one, Watterson’s $\theta$ ($\theta _W$), is based on the number of polymorphic sites. Tajima’s D obtains negative values in the proximity of a selective sweep, since $\pi$ decreases with both high- and low-frequency derived variants, while $\theta _W$ remains unaffected.

In 2000, Fay and Wu [11] proposed a new statistic, the well-known Fay and Wu’s H, which obtains low values in regions where high-frequency derived variants are overrepresented. To distinguish between high- and low-frequency derived variants, Fay and Wu’s H relies on information derived from an outgroup species. The ancestral state is considered to be the one that is common between the ingroup and the outgroup. Additionally, Fay and Wu [11] invented a new unbiased estimator for $\theta$, named $\theta _H$, which assumes high values in regions with overrepresented high-frequency derived variants. The H statistic is defined as the difference between $\pi$ and $\theta _H$, and as such it becomes significantly negative in the proximity of a beneficial mutation. Since a backmutation will result in the incorrect inference of the derived polymorphic state, Fay and Wu’s H requires the probability of mis-inference to be incorporated in the construction of the null distribution of the statistic. In 2006, Zeng et al. [50] improved the H statistic by adding the variance of the statistic in the denominator, thus scaling H by the variance of the statistic.

Depaulis and Veuille [51] introduced two neutrality tests that rely on haplotypic information. The first summary statistic, K, is simply the number of distinct haplotypes in the sample, assuming low values in the proximity of the beneficial mutation. The second test measures haplotype diversity, denoted by H (or DVH, Depaulis and Veuille H, to be distinguished from Fay and Wu’s H). DVH is calculated as $DVH = 1 - \sum _{i=1}^K p_i^2$, where $p_i$ is the frequency of the* i*th haplotype. Both the DVH and the K summary statistics are conditioned on the number of polymorphic sites, *s*, which yields the construction of the null (neutral) distribution of the statistic rather problematic. Depaulis and Veuille simulated data using a fixed number of polymorphic sites *s*, and without conditioning on the coalescent trees. This approach is incorrect because the number of polymorphic sites is a random variable that follows a Poisson distribution, and it is determined by the total length of the (local) coalescent tree and the mutation rate. Thus, to construct the null distribution of the statistic, a two-step approach is required: first, a coalescent tree is generated according to the demographic model and mutations are placed randomly on its branches (this step can be achieved using Hudson’s ms [52]), and second, a rejection process is applied in order to condition on the number of polymorphic sites *s*, during which only the simulations that produced *s* segregating sites are kept while the rest are discarded.

Typically, summary statistics are applied on whole genome data following a sliding-window approach, which allows inexpensive computations on large datasets for those statistics used as neutrality tests. However, two problems exist with the use of summary statistics as neutrality tests. The first problem is that the window size is fixed, which, regardless of the way it is measured, i.e., either as number of SNPs or as number of base pairs, it can be of critical importance for the acceptance or rejection of the null hypothesis. For example, it is possible to not reject neutrality when using Tajima’s D on 1-kb windows, while rejecting neutrality when using the same summary statistic on 2-kb windows. More advanced tests, such as SweepFinder/SweepFinder2, SweeD, and OmegaPlus implement variable-sized windows (see below). While evaluating windows of varying sizes does not solve the problem completely, due to the inevitable existence of lower and upper bounds for the window sizes, such tests are more robust to the window size parameter. The second problem, which is common for most neutrality tests, is that they are not robust to demographic changes of the population. For instance, Tajima’s D can assume negative values in a population expansion scenario as well as locally in genomic regions under a bottleneck scenario. It also becomes negative in genomic regions that have experienced purifying selection. Fay and Wu’s H can become negative in demographic models that increase the high-frequency derived variants. Such demographic models include gene flow [53] or sampling from one deme that is part of a metapopulation [54] (Pavlidis, unpublished data).

### Detecting sweeps in subgenomic regions

In addition to summary statistics, which due to low computational costs are highly suitable for scanning whole genomes, various stand-alone software implementations have also been released in the previous years, with initial releases focusing mostly on the analysis of subgenomic regions with limited number of SNPs, due to increased computational requirements.

#### Kim and Stephan test [30]

The Kim and Stephan test [30] (known also as CLR test), used the results of Fay and Wu [11] to obtain the probability to observe a mutation of certain frequency *p*, at some distance from the location of the selective sweep. Under a selective sweep model, only low and high frequency derived alleles have non-zero probabilities, whereas under a neutral model, the probability to observe a mutation of certain frequency is given by the standard neutral SFS. Then, a Composite Likelihood Ratio test (CLR) is performed. High CLR values denote a candidate region for a selective sweep. To obtain a threshold value for the CLR, simulations should be performed under a reference demographic model (without selection). The Kim and Stephan test can be only applied on subgenomic data.

#### Pavlidis et al. [55]

The detection approach proposed by Pavlidis et al. [55] relies on a machine-learning paradigm to detect selective sweeps in candidate subgenomic regions. This approach implements a support vector machine (SVM) classifier to separate neutral datasets from datasets with selection and demography. SVM classifiers, and in general supervised machine learning approaches, require a training phase, where the algorithm “learns” to separate neutral from selection scenarios based on concrete simulated examples, either neutral or selected ones. In the training phase, neutral models incorporate the demographic model, whereas selection models incorporate both the demographic model and selection. One problem that arises from such an approach is that a multitude of models might exist for the models with selection (e.g., time of the onset of beneficial mutation and selection coefficient). Pavlidis et al. [55] used a mixture of selection models with various selection coefficients and various onset times of the beneficial mutation. The method evaluation revealed satisfying results, but the required training phase of the SVM prevented the application of this approach at a full-genome scale, due to prohibitively large execution times.

### Detecting sweeps in whole genomes

The advent of Next Generation Sequencing (NGS) paved the way for the analysis of whole genomes at different geographic locations and environmental conditions, and revealed a need for more efficient processing solutions in order to handle the increased computational and/or memory requirements generated by large-scale NGS data. While typical summary statistics are generally suitable for NGS data, they are applied on fixed-size windows, and as such they do not provide any insight on the extent of a selective sweep. More advanced methods that rely on the CLR test (e.g., SweepFinder [16], SweepFinder2 [56], and SweeD [17]) or on patterns of LD (e.g., OmegaPlus [18, 57]), perform a window-size optimization approach that provides information on the genomic region affected by a selective sweep at the cost of increased execution times. The aforementioned methods have been widely used to detect recent and strong positive selection in a variety of eukaryotic or prokaryotic organisms, such as human [16, 58, 59], *D. melanogaster* [60–63], lizards [64], rice [65], butterflies [66], and bacteria [67].

#### SweepFinder

In 2005, Nielsen et al. [16] released SweepFinder, an advanced method to detect selective sweeps that relies on information directly derived from the SFS. SweepFinder implements a composite likelihood ratio (CLR) test, with the numerator representing the likelihood of a sweep at a given location in the genome, and the denominator accounting for the neutral model. An important feature of SweepFinder is that neutrality is modeled based on the empirical SFS of the entire dataset. All SNPs are considered independent, therefore allowing the likelihood score per region for the sweep model to be computed as the product of per-SNP likelihood scores over all SNPs in a region. SweepFinder was among the first software releases with the capacity to analyze whole genomes via a complete and standalone implementation.

SweepFinder can process small and moderate sample sizes efficiently. However, the source code does not include support for a large number of sequences, yielding analyses with more than 1027 sequences numerically unstable due to unhandled floating-point underflows [17]. Additionally, SweepFinder only executes sequentially, therefore not exploiting all the computational resources in modern x 86 processors (e.g., multiple cores and intrinsic instructions).

#### SweeD

Pavlidis et al. [17] released SweeD (**Swee**p **D**etector), a parallel and optimized implementation of the same CLR test as SweepFinder. SweeD can parse various input file formats (e.g., Hudson’s ms, FASTA, and the Variant Call Format) and provides the option to employ a user-specified demographic model for the theoretical calculation of the expected neutral SFS. Pavlidis et al. [17] showed that sweep detection accuracy increases with an increasing sample size, and altered the mathematical operations for the CLR test implementation in SweeD to avoid numerical instability (floating-point underflows), allowing the analysis of datasets with thousands of sequences.

The time-efficient analysis of large-scale datasets in SweeD is mainly due to two factors: (a) parallel processing using POSIX threads, and (b) temporary storage of frequently used values in lookup tables. Additionally, SweeD relies on a third-party library for checkpointing (Ansel et al. [68]) to allow resuming long-running analyses that have been abruptly interrupted by external factors, such as a power outage or a job queue timeout.

#### SweepFinder2

**Fig. 4:**
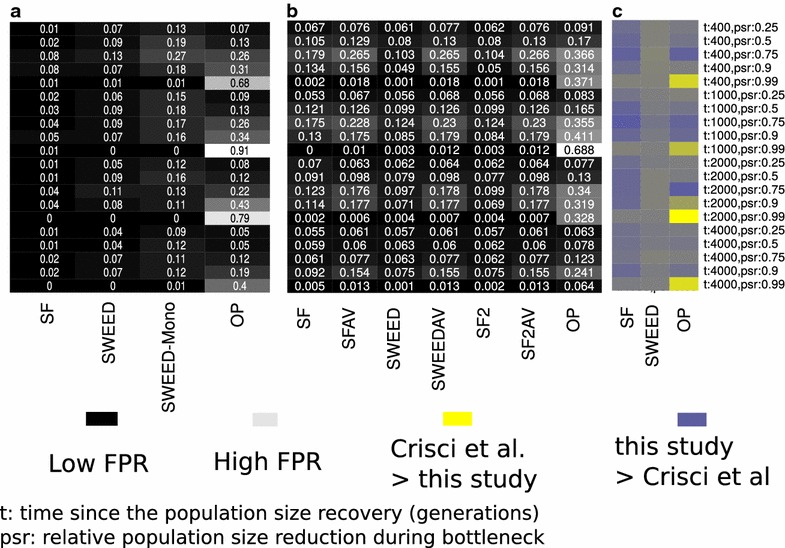
False positive rates for the selective sweep detection process under various algorithms and demographic models. Demographic models consist of bottlenecks and are characterized by two parameters: *t* is the time in generations since the recovery of the populations, and *psr* the relative population size reduction during bottleneck. Prior to the bottleneck, the population size equals to the present-day population size. We show the results from the study of Crisci et al. [15] (**a**), our analysis in the current study (**b**) and the difference between** a** and** b** (**c**). Note that Crisci et al. studied SweepFinder (SF), SweeD (SWEED), SweeD with monomorphic (SWEED-Mono) and OmegaPlus (OP). In the current work, we studied SweepFinder (SF), SweepFinder with average SFS (SWEEDAV), SweeD (SWEED), SweeD with average SFS (SWEEDAV), SweepFinder2 (SF2), SweepFinder2 with average SFS (SF2AV), and OmegaPlus. Thus, in** c** we show only results from the common tools (SF, SWEED, OP). In** a** and** b**, the darker a cell, the lower the false positive rate. In** c**,* yellow* denotes that Crisci et al. report higher false positive rate than this study, while* blue* denotes that the reported false positive rate by Crisci et al. is lower

More recently, DeGiorgio et al. [56] released SweepFinder2. SweepFinder2 uses the statistical framework of SweepFinder, and additionally it takes into account local reductions in diversity caused by the action of negative selection. Therefore, it provides the opportunity to distinguish between background selection and the effect of selective sweeps. Thus, it exhibits increased sensitivity and robustness to background selection and mutation rate variations. Besides the ability to account for reductions in the diversity caused by background selection, the implementation of SweepFinder2 is very similar to SweepFinder. However, there exist code modifications that increase the stability of SweepFinder2 on the calculation of likelihood values. Using simulated data with constant mutation rate and in the absence of negative selection, SweepFinder2 results in more similar to SweeD than to the initial SweepFinder implementation (see Fig. 4).

#### OmegaPlus

In 2012, Alachiotis et al. [18] released a high-performance implementation of the $\omega$-*statistic* [12] for the detection of selective sweeps by searching for a specific pattern of LD that emerges in the neighborhood a recently fixed beneficial mutation. The $\omega$-*statistic* assumes a high value at a specific location in the genome, which can be indicative of a potential selective sweep in the region, if extended contiguous genomic regions of high LD are detected on both sides of the location under evaluation, while the level of LD between the high LD regions remains relatively low.

OmegaPlus evaluates multiple locations along a dataset following an exhaustive per-region evaluation algorithm which was initially introduced by Pavlidis et al. [55]. The algorithm by Pavlidis et al. [55] required large memory space for the analysis of many-SNP regions and exhibited increased complexity, yielding the analysis of regions with thousands of SNPs computationally unfeasible. OmegaPlus introduced a dynamic programming algorithm to reduce the computational and memory requirements of the exhaustive evaluation algorithm, enabling the efficient analysis of whole-genome datasets with millions of SNPs. OmegaPlus exhibits a series of four different parallelization alternatives [57, 69] for the distribution of computations to multiple cores to overcome the load balancing problem in selective sweep detection due to the difference in SNP density between regions in genomes.

#### MFDM test

In 2011, Li et al. [70] presented a neutrality test that detects selective sweep regions using the Maximum Frequency of Derived Mutations (MFDM), which is a paramount signature of a selective sweep. According to [70], the MFDM test is robust to processes that occur in a single and isolated population. This is because there is no demographic scenario in single and isolated populations that generates a non-monotonic SFS and increases the amount of high-frequency derived variants. Thus, at least in theory, the test is robust to demographic models, such as bottlenecks, when they occur in isolated populations.

There are, however, four severe problems regarding the robustness of the test, which broadly apply to other tests of neutrality as well: (a) although bottlenecks generate monotonic *average* SFSs, certain genomic regions may locally exhibit increased amounts of high-frequency derived variants, even in the absence of positive selection, (b) high-frequency derived variants are a signature of selective sweeps in constant populations but it is not known whether and how they will be affected by the combined action of selection and demography, (c) in populations that exchange migrants with other demes (non-isolated), the frequency of high-frequency derived variants may increase (e.g. [53]), and (d) backmutations (in general, the violation of the infinite site model) may also increase the amount of high-frequency derived variants (Pavlidis, unpublished data).

## Evaluation

The aforementioned software tools (SweepFinder, SweepFinder2, SweeD, and OmegaPlus, see Table 1) have been independently evaluated by two studies: Crisci et al. [15] studied the effect of demographic model misspecification on selective sweep detection, while Alachiotis and Pavlidis [69] conducted a performance comparison in terms of execution time for various dataset sizes and number of processing cores. We summarize these results in the following subsections and partially reproduce the FPR evaluation analysis by Crisci et al. [15], including SweepFinder2. Besides demography, we also demonstrate how the number of polymorphic sites affects the outcome of SFS-based and LD-based neutrality tests. Note that, the iHS software [19] is also considered in both studies, but is not included in the following comparison summary due to its different scope: iHS detects ongoing sweeps relying on extended haplotypes, and not complete sweeps.

**Table 1:** List of software tools for selective sweep detection

	Method	Implementation	Availability (source code, web service)
SweepFinder (2005)	SFS	Sequential	http://people.binf.ku.dk/rasmus/webpage/sf.html, –
OmegaPlus (2012)	LD	Parallel	https://github.com/alachins/omegaplus , http://pop-gen.eu
SweeD (2013)	SFS	Parallel	https://github.com/alachins/sweed , http://pop-gen.eu
SweepFinder2 (2016)	SFS	Sequential	http://www.personal.psu.edu/mxd60/sf2.html, –

### Detection accuracy

Crisci et al. [15] calculate the FPR for the neutrality tests using the following pipeline: (1) simulations from equilibrium models using Hudson’s ms [52] and constant number of SNPs. This set of simulations is used only for the determination of the thresholds for the tools; (2) simulations using sfscode [71] (constant or bottlenecked population). These data are called empirical datasets, and are used for the estimation of the FPR; (3) execution of the neutrality tests on the empirical datasets. The FPR is estimated by assigning each empirical dataset to a threshold value from an equilibrium model with similar number of SNPs. Note that, such an approach differs from the approach that has been followed by other studies (e.g. [72, 73]), where the null model is specified by the inferred neutral demographic model. Specifying the null model by the inferred neutral demographic model controls efficiently for the FPR. Thus, Crisci et al. effectively studied how demographic model misspecification affects the FPR. Another major difference between the approach followed by Crisci et al. and other studies is that, for the SFS-based methods (SweepFinder, SweeD), Crisci et al. calculate the neutral (or *prior-to-sweep*) SFS using the candidate region itself (here 50 kb), instead of the average SFS on a chromosome-wide scale. Even though the first approach might have a lower FPR, the later is more powerful to detect selective sweeps: when the neutral SFS is calculated by a small genetic region that potentially includes a sweep, the affected (by the sweep) SFS is assumed to represent neutrality. Thus, the CLR test will assume lower values. For neutral equilibrium models, i.e., constant population size, they find that the FPR for SweepFinder ranges from 0.01 to 0.18, depending on the mutation and recombination rate: the lower the mutation and recombination rates the higher the FPR of SweepFinder. The FPR for SweeD ranges between 0.04 and 0.07. For OmegaPlus, the FPR ranges between 0.05 and 0.07. In general, the FPR for all tools is low when the demographic model is at equilibrium.

When the assumption of an equilibrium population is violated and the empirical datasets are derived from bottlenecked populations, the FPR increases. Such an increase of the FPR is more striking when the average SFS of the empirical dataset is used to represent the SFS of the null model. The reason for such an increase is that bottlenecked datasets show great variance of the SFS from a region to another. Thus, even though, on average, a bottlenecked population will have a monotonically decreasing SFS [74], there might be regions that show an excess of high-frequency and low-frequency derived variants, and thus they mimic the SFS of a selective sweep.

Interestingly, Crisci et al. report low FPR for SweepFinder and SweeD. For OmegaPlus, the FPR they report is high for the very severe bottleneck scenario, where the population size has been reduced by 99%. For SweepFinder and SweeD, the FPR ranges between 0 and 0.08, and 0 and 0.13, respectively. For OmegaPlus, they report FPR between 0.05 and 0.91. We repeated the analysis of Crisci et al. for SweeD, SweepFinder, and OmegaPlus, including also SweepFinder2. Furthermore, we have included execution results of SweepFinder, SweeD and SweepFinder2 using the average SFS instead of the regional SFS. We used Hudson’s ms for *all* simulations, whereas Crisci et al. have used sfs_code for the empirical simulated data. In general, our results are comparable to Crisci et al., but we report higher FPR than Crisci et al. A notable exception is the case of OmegaPlus in the severe bottleneck case, where our FPR are considerably lower. Perhaps this is due to the simulation software, as we used Hudson’s ms (coalescent) simulator, and Crisci et al. used sfs_code (forward). FPR results are shown in Fig. 4.

**Fig. 5:**
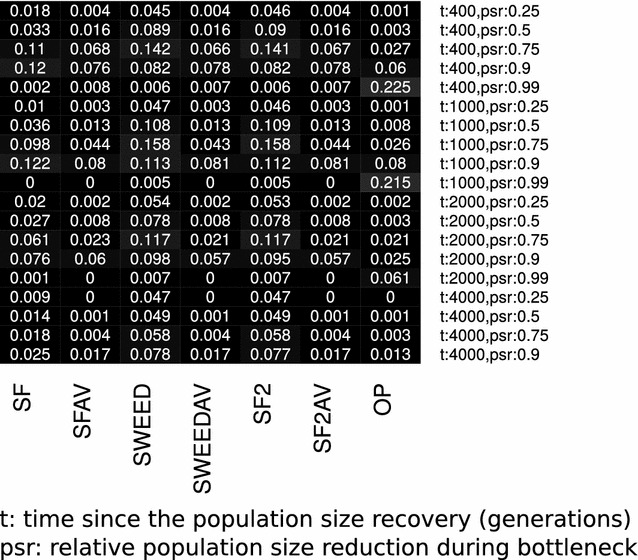
False positive rates for the selective sweep detection process under various algorithms and demographic models when the demographic model used for the construction of the threshold value is a bottleneck model instead of an equilibrium model. To compute *all* threshold values, we have used the bottleneck model characterized by a population recovery at time $t=1000$ generations, and bottleneck population size reduction by 0.90. The duration of the bottleneck was 4000 generations. FPR values have been reduced considerably compared to the case that the equilibrium model was used for the calculation of the threshold values (Fig. 4)

Since FPR is considerably increasing when a false model (e.g., equilibrium) is used to construct the null hypothesis, we repeated the aforementioned analysis using a bottleneck demographic model. Using a bottleneck demographic model for the construction of the null hypothesis reduces the FPR to very low values (Fig. 5). Here, we have used the bottleneck model characterized by a population size reduction of 0.99, a recovery time of 1000 generations, and bottleneck duration of 4000 generations, even though empirical datasets were composed by additional models. The ancestral population size was equal to the present day population size.

Regarding the True Positive Rate (TPR), Crisci et al. report that under strong selection in an equilibrium population ($2 N_e s=1000,$ where *s* is the selection coefficient), TPR for SweepFinder and SweeD is moderate and ranges between 0.32 and 0.34. For OmegaPlus, TPR is higher and equals to 0.46. For weaker selection ($2 N_e s=100$), OmegaPlus also remains the most powerful tool to detect selective sweeps. For selective sweep models in bottlenecked populations, OmegaPlus outperforms SFS-based methods and it is the only test studied by Crisci et al. able to detect selective sweeps. Finally, regarding recurrent hitchhiking event (RHH), OmegaPlus reports higher values of TPR.

### Execution time

The performance comparisons conducted by [69] aimed at evaluating the effect of the number of sequences and SNPs on execution time, as well as the capacity of each code to employ multiple cores effectively to achieve faster execution. Table 2 shows execution times on a single processing core for different dataset sizes, ranging from 100 sequences to 1000 sequences, and from 10,000 SNPs up to 100,000 SNPs. Additionally, the table provides (in parentheses) how many times faster are SweeD and OmegaPlus than SweepFinder.

**Table 2:** Comparison of execution times (in seconds) for different dataset sizes (Fomat: D-number of sequences-number of SNPs) on a single processing core [69]

	D-10^2^–10^4^	D-10^2^–10^5^	D-10^3^–10^4^	D-10^3^–10^4^
SweepFinder	540 (1×)	4138 (1×)	132,938 (1×)	135,996 (1×)
SweeD	125 (4.3×)	1169 (3.5×)	283 (469×)	1345 (101×)
OmegaPlus	6 (90×)	652 (6.4×)	7 (18,991×)	753 (180×)

The comparison between SweepFinder and SweeD is the most meaningful one since both tools implement the same floating-point-intensive CLR test based on the SFS, thus requiring the same type and amount of arithmetic operations. The significantly faster execution of OmegaPlus on the other hand, which relies on LD, is attributed to the fact that a limited number of computationally intensive floating-point operations are required, with the majority of operations being performed on integers, such as the enumeration of ancestral and derived alleles.

The execution times in Table 2 refer to sequential execution. Multiple cores can be employed by SweeD and OmegaPlus, achieving speedups that vary depending on the number of sequences and SNPs. The parallel efficiency of SweeD decreases with an increasing sample size, whereas the respective parallel efficiency of OmegaPlus increases. As the number of SNPs increases, both SweeD and OmegaPlus exhibit poorer parallel efficiency, which is attributed to load balancing issues that arise with an increasing variance in the SNP density along the datasets.

## Detection of soft sweeps

The methods and approaches reviewed in this manuscript are appropriate for the detection of complete selective sweeps that originate from a *new* beneficial variant. Such selective sweeps are called ‘hard’ selective sweeps. If positive selection acts, however, on variation already segregating in the population, or if multiple beneficial alleles arise independently, the models of ‘hard’ selective sweeps do not apply. Hermisson and Pennings [75–77] coined the term ‘soft’ selective sweeps to describe such alternative models of positive selection. Soft sweeps have been documented in sticklebacks [78] and beach mice [79]. In humans, several cases of selection from standing genomic variation have been reported [80–82]. The detection of soft sweeps is notably more challenging than the detection of ‘hard’ selective sweeps, because soft selective sweeps do not affect linked neutral polymorphism to the same extent as hard selective sweeps.

Ferrer-Admetlla et al. [83] described a haplotype-based statistic, called $nS_L$: number of Segregating sites by Length, designed to detect both soft and hard selective sweeps. $nS_L$ uses phased data and it calculates the ratio of haplotype homozygosity for the derived and ancestral state alleles. Such an approach is also taken by the iHS statistic [19]. In contrast to iHS, however, $nS_L$ measures the length of a segment of haplotype homozygosity between a pair of haplotypes in terms of number of mutations in the remaining haplotypes, in the same region. Therefore, a genetic map is not required and $nS_L$ is more robust to recombination and mutation rate fluctuations.

Garud et al. [84] developed several haplotype homozygosity statistics to capture the increase of haplotype homozygosity observed in both hard and soft sweeps. According to [84], haplotype homozygosity is defined as $H1 = \sum _1^n p_i^2$, for *n* distinct haplotypes. The *H*1 statistic is equivalent to the haplotype heterozygosity statistic of Depaulis and Veuille [51] (see above), and assumes high values in a hard sweep case because heterozygosity in a region affected by a hard selective sweep is dramatically decreased. However, for soft selective sweeps, the power of *H*1 is expected to decrease because additional haplotypes are present. Two additional statistics were developed by Garud et al. [84], which mainly facilitate the detection of soft sweeps: (a) the *H*12 statistic, defined as: $H12 = (p_1 + p_2)^2 + \sum _{i>2}^n p_i^2 = H1 + 2 p_1 p_2$, in which the frequencies of the first and the second most common haplotypes are combined into a single frequency, and (b) the *H*123 statistic, in which the frequencies of the three most common haplotypes are combined into a single measurement. Since the frequencies of the most abundant haplotypes are separated into an additional value, the values of *H*12 and *H*123 are considerably increased in the proximity of a soft sweep.

Soft selective sweeps have attracted attention in recent literature mainly because they are not restricted by the limited amount of new beneficial mutations (in contrast to hard selective sweeps), and because of the limited amount of hard selective sweep patterns found in natural populations (especially human [85] and *D. melanogaster* [84]). It has been pointed recently by Jensen [86], however, that such an enthusiasm for soft selective sweeps may be unfounded, based on both theoretical and experimental insights. Jensen [86] stresses as a potential reason for the limited amount of selective sweeps detected in natural populations the reduced power of existing tests to detect hard selective sweeps in the presence of complex demographic models. As argued above, such a lack of power may spring from the fact that under certain demographic models we are forced to increase the detection threshold in order to control the FPR. Therefore, several true targets are also discarded. Additionally, selective sweep models are designed assuming a constant, equilibrium population. Different demographic models combined with positive selection may however generate different patterns of selective sweeps, though have remained unexplored until now. Therefore, it becomes clear that under non-equilibrium demographic models and/or violations of the hard selective sweep model, our ability to detect selection decreases. This, however, does not mean that selection is absent: absence of evidence does not necessarily imply evidence of absence.

## Discussion

### Overinterpretation of results and storytelling

Identifying genomic regions that have undergone recent and strong positive selection is an important challenge of modern evolutionary biology. Neutral evolutionary processes, such as random genetic drift enhanced by population size changes and/or gene flow, increase the rate of false positives and make it more challenging to detect genomic regions which have been targeted by positive selection. Frequently, additional validity of results is provided by the fact that loci identified by selective sweep scans ‘make sense’. Pavlidis et al. [87] showed that such an approach of perceiving an increased validity of results, simply because they make sense can be dramatically misleading. They designed a simple simulation experiment, in which a neutrally evolved X-chromosome of *D. melanogaster* is scanned for selective sweeps. Then, they performed a literature mining for the (by definition false positive) identified selective sweep targets. They showed that by means of gene ontology it would make perfect sense to identify such targets even though they are false positives. The study by Pavlidis et al. [87] showed that interpretation of the results should be treated very carefully and overinterpretation should be avoided.

### Combining methods to decrease the false positive rate

**Fig. 6:**
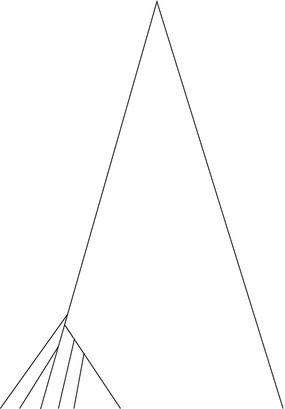
An unbalanced genealogy with several short external branches can generate extreme values for a multitude of neutrality tests

To increase the validity of selective sweep scans, analyses typically consist of a multitude of neutrality tests. The rationale is that ‘the more tests agree on an outcome, e.g., selection, the more plausible this outcome is’. The problem with this, however, is that the outcome of different neutrality tests are usually correlated, since they depend profoundly on the underlying coalescent tree. Consider a neutrally evolved genomic region that is characterized by an exceptional ‘sweep-like’ collection of coalescent trees. Several neutrality tests will give a good signal for a selective sweep in this region. For instance, assume a set of unbalanced trees, such as those shown in Fig. 6, where all lineages except for one coalesce relatively fast on one side of the tree. Tajima’s D assumes extreme values because of the skewed SFS. The same is true for SweeD and SweepFinder. Furthermore, since the tree is unbalanced with long internal branches, LD is increased locally. The number of polymorphic sites might be reduced since the total tree length is reduced. Thus, independently applying several neutrality tests and then showing that several of them reject neutrality (or showing only those that reject neutrality) should be avoided. A better practice is to combine the tests in a unified framework and not independently. For example, [55, 88, 89] used supervised learning algorithms and several neutrality tests (variables) to classify genomic regions as either neutral or selected. Any correlation between the variables is incorporated implicitly in the learning algorithms and does not affect the accuracy of the classifier. Since, however, a large number of simulations is typically required for the execution of the learning algorithms, the running time of such approaches increases considerably.

### The need for high performance

Driven by the advent of DNA sequencing, several projects have focused on sequencing whole genomes from various species in the past years. This has led to the discovery of thousands of new SNPs and the availability of a plethora of datasets that are suitable for population genetics analyses. As more genomes are being sequenced, contributing to the increasing dataset sizes, the computational demands for the respective analyses increase as well. This poses a challenge to existing and future software tools as High Performance Computing (HPC) techniques are becoming a prerequisite for conducting large-scale analyses.

Reducing execution times and enabling processing of large-scale datasets on limited hardware resources, such as off-the-shelf workstations, requires source codes to abide by several basic HPC principles. For instance, understanding how memory accesses affect performance, or which scheduling/communication strategy among multiple cores is the most efficient for a particular task, can substantially reduce execution times by allowing the software to utilize the hardware resources in current x 86 processors in the most effective way. With Moore’s law being continued in the form of an increasing number of cores per processor and an increasing width for vector registers^1^, not employing multithreading^2^ and/or vector intrinsic instructions in newly developed tools can lead to significant underutilization of processors.

However, although optimization techniques such as kernel vectorization have the potential to accelerate processing, the nature of operations and the computational demands of the target task for performance improvement need to be carefully examined. For instance, a recent study [90] revealed that in order to achieve high-performance for large-scale LD computations that comprise thousands of sequences and SNPs, vector intrinsics must be avoided. This is due to the fact that the computational bottleneck in LD-based analyses for large sample sizes is the enumeration of ancestral and derived alleles in SNPs. This operation is efficiently implemented via the use of an intrinsic population count command, which however operates only on regular registers, i.e., 32- or 64-bit words. Deploying vector intrinsics for LD leads to poorer performance due to increased data preparation times (storing and retrieving words in vector registers).

In addition to software-level optimizations for faster completion of bioinformatics analyses, a variety of hardware-accelerated solutions have also been proposed in the previous years. Hardware platforms, such as Graphics Processing Units (GPUs) and Field Programmable Gate Arrays (FPGAs), have been widely targeted for the acceleration of large-scale analyses, and a variety of bioinformatics algorithms have been successfully ported on these architectures, from sequence alignment kernels [91] and phylogenetic tree scoring functions [92, 93] to large-scale LD computations [90] and epistasis detection in Genome Wide Association Studies [94].

## Conclusions

Detecting recent and strong positive selection is a fascinating challenge of modern population genetics. In this manuscript, we conducted a survey of approaches, methods, and software packages that can be used to pinpoint the genomic regions where positive selection has operated recently. A multitude of approaches can be used for such a purpose, aiming at capturing genomic selective sweep signatures. Regarding computational efficiency, selective sweep detection methods range from computationally inexpensive summary statistics to complete software releases with higher computational and memory demands, that offer greater flexibility (variable window size) and are able to estimate selection-related parameters (e.g. selection strength, size of the genomic region affected by the selective sweep). Despite the progress in the development of approaches to detect selective sweep, scanning for selective sweeps remains a challenging task mainly because of the confounding effect of demography. Thus, even though demography affects the whole genome, its effect is not homogeneous. In contrast, demography, especially bottlenecks, can generate local SNP patterns in the genome that are similar to those patterns generated by positive selection. In a whole-genome analysis it is extremely challenging, if not unfeasible, to separate such pseudo-selective sweep signatures from real selective sweeps. We emphasize that further research is needed to successfully detect selective sweeps within a non-equilibrium population (e.g., when the population size changes) because the respective sweep patterns may differ from the expected signatures that are detected by existing software tools. Moreover, over-interpretation of the results, in terms of Gene Ontology, should be avoided. Understanding the strengths and limitations of the methods and tools is crucial to avoid unnecessarily long execution times and/or misled conclusions.

## Footnotes

1 Most commodity processors support vector processing, i.e., single instructions that operate on one-dimensional arrays of data that are stored in vector registers.

2 Multithreading is a coding technique that enables the software to deploy multiple cores per processor for parallel processing.
